# Discovery of Novel Biomarkers of Therapeutic Responses in Han Chinese Pemetrexed-Based Treated Advanced NSCLC Patients

**DOI:** 10.3389/fphar.2019.00944

**Published:** 2019-08-23

**Authors:** Xiaoqing Zhang, Di Zhang, Lihua Huang, Guorong Li, Luan Chen, Jingsong Ma, Mo Li, Muyun Wei, Wei Zhou, Chenxi Zhou, Jinhang Zhu, Zhanhui Wang, Shengying Qin

**Affiliations:** ^1^Department of Pharmacy, The International Peace Maternity and Child Health Hospital, School of Medicine, Shanghai Jiao Tong University, Shanghai, China; ^2^Life Science College, Anhui Medical University, Hefei, China; ^3^Bio-X Institutes, Key Laboratory for the Genetics of Developmental and Neuropsychiatric Disorders (Ministry of Education), Shanghai Jiao Tong University, Shanghai, China; ^4^Department of Oncology, Shanghai Pulmonary Hospital, Tong Ji University, Shanghai, China; ^5^School of Life Sciences, Shandong Normal University, Jinan, China; ^6^Department of General Surgery, Luoyang Central Hospital Affiliated to Zhengzhou University, Luoyang, China; ^7^The Third Affiliated Hospital, Guangzhou Medical University, Guangzhou, China

**Keywords:** pemetrexed, non–small cell lung cancer, adverse drug reactions, therapeutic effects, single nucleotide polymorphism, biomarker

## Abstract

Pemetrexed, one of the most commonly used drugs in advanced non–small cell lung cancer (NSCLC) therapies, often leads to various therapeutic responses in patients. These therapeutic responses to pemetrexed, including adverse drug reactions (ADRs) and its intended therapeutic effects, have been demonstrated to be highly individual-specific. Such difference in therapeutic responses across individuals may be caused by the unique genetic variations in each patient. However, only a few pemetrexed-based studies have been performed using Han Chinese patients. In this study, we aimed to identify genetic signatures of therapeutic responses of pemetrexed-based treatment using 203 Han Chinese patients with advanced NSCLC. All the participants received two different types of therapies: 1) treatment with only pemetrexed and 2) treatment with both pemetrexed and platinum (mainly cisplatin and carboplatin). We then performed a genetic association analysis on 16 selected single-nucleotide polymorphisms (SNPs) in 7 genes using these 2 groups. The analysis of patients receiving only pemetrexed suggests that the SNP rs1051298 on the *SLC19A1* gene (c.*746C > T) increased the risk of all ADRs (collected all types of ADRs) in different cycles of pemetrexed therapy [1-2 cycles: *P* = 0.0059, odds ratio (OR) = 3.143; 1-4 cycles: *P* = 0.0072, OR = 2.340; 1-6 cycles: *P* = 0.0071, OR = 2.243]. This influence of rs1051298 is particularly significant in terms of liver injury (1-4 cycles: *P* = 0.0056, OR = 3.863; 1-6 cycles: *P* = 0.0071, OR = 3.466). In all the patients, including patients who received both pemetrexed and platinum, SNP rs1801133 on the *MTHFR* gene (665C > T) was found to be significantly associated with hematological ADRs in 1 to 2 cycles (*P* = 0.0079, OR = 3.566). Additionally, we discovered that SNP rs12995526 (c.815-102T > C) in the *ATIC* gene and SNP rs11545077 (c.91G > T) in the *GGH* gene were associated with both ADRs and therapeutic effects. In summary, our study identified several potential biomarkers that were significantly associated with ADRs and therapeutic effects of pemetrexed-related treatments using Han Chinese patients. Our discoveries will provide important clues for personalized pemetrexed-based treatment design for Han Chinese NSCLC patients in the future.

## Introduction

Lung cancer is a fatal disease that has become one of the most common tumors around the globe in recent years (Singh et al., 2017). Non–small cell lung cancer (NSCLC), the most frequent lung cancer of all types (Bradbury et al., 2018), is often treated with pemetrexed. Pemetrexed was often used in combination with platinum compounds in the treatment of NSCLC to reduce the pain and extend the life of the patient (Okamoto et al., 2013). It is a multitargeted antifolate medicine that has been widely used in maintenance treatment for patients with advanced NSCLC (Kato et al., 2014). It reduces the proliferation of cancer cells, thus inhibiting further progression of NSCLC in the patient’s body (Hu et al., 2014).

While pemetrexed extends life and improves life quality in certain groups of NSCLC patients, it sometimes leads to various severe adverse drug reactions (ADRs) and shorter survival time and worsens the health condition of patients (Yee et al., 2010; Corrigan et al., 2014). Severe ADRs refer to unwanted adverse effects, including skin rash, hematological toxicity, gastrointestinal toxicity, hepatotoxicity, and nephrotoxicity (Perez-Ramirez et al., 2016). Adverse drug reactions and therapeutic effects of the drug are usually coexistent in treated patients (Ruan et al., 2016; Wang et al., 2018). Furthermore, it is inevitable for pemetrexed-treated NSCLC patients to eventually develop resistance to this drug, resulting in its decreased therapeutic effects. Most current solutions of ADRs to pemetrexed only reduce the pain of ADRs resulting from the treatment. Previous researches suggest that the tolerability of ADRs and therapeutic effects in pemetrexed-based therapies are highly individual-specific, which could be explained by the genetic association between each patient and his/her ADRs to pemetrexed-based chemotherapy (Adjei et al., 2010b; Corrigan et al., 2014).

Multiple genes have been found to impact the ADRs and therapeutic effects of pemetrexed, such as *SLC19A1*. *SLC19A1* plays a critical role in the process of intracellular uptake of pemetrexed. This gene encodes folate carrier protein 1 (RFC1), which is highly involved in intracellular uptake of folate (Yee et al., 2010). It has been reported that RFC1 affects the transport process of pemetrexed *in vivo* (Westerhof et al., 1995). In addition to folate absorption, RFC1 impacts folate homeostasis of mammalian cells and is down-regulated in response to folate deficiency (Ifergan et al., 2008). Single-nucleotide polymorphisms (SNPs) in the *SLC19A1* gene may alter the molecular transportation process of pemetrexed. Therefore, polymorphism in *SLC19A1* may exert significant influence on the therapeutic responses of pemetrexed-based chemotherapy in each patient (Alnatsha et al., 2018).

In addition to *SLC19A1*, there are other important genes that contribute to the individual difference of ADR occurrences and therapeutic effects of pemetrexed(Adjei et al., 2010a; Woo et al., 2015). These genes are usually the ones that are actively involved in folate transport and metabolic pathway, including folylpolyglutamate synthase (*FPGS*), gamma-glutamyl hydrolase (*GGH*), methylenetetrahydrofolate reductase (*MTHFR*), thymidylate synthase (*TS*), dihydrofolate reductase (*DHFR*), and 5-aminoimidazole-4-carboxamide-ribonucleotide formyltransferase (*ATIC*). The *GGH* and *MTHFR* genes play a significant part in metabolic pathway of pemetrexed (Fukuda et al., 2016). *GGH* removes these glutamic acid residues generated by the metabolism of pemetrexed, so that the activity of drugs is reduced. At last, *ATIC* gene represses the process of methotrexate, which is involved in the metabolic pathway of pemetrexed. Pemetrexed targets the enzyme products of these genes, such as *TS*, *DHFR*, and *ATIC*, which are engaged in both pyrimidine or purine synthesis and apoptosis of cells (Wang et al., 2014). Upon entering the cytoplasm, pemetrexed is rapidly transformed into the product of polyglutamate under *FPGS* gene regulation (Habeck et al., 1995).

A great number of researches have been conducted to study the prognosis, ADRs, and therapeutic effects to pemetrexed-based therapy in the past decades (Li et al., 2012; Wang et al., 2013; Liu and Wu, 2015). However, there are very few reports focused on the biomarkers of ADRs and therapeutic effects to pemetrexed-based chemotherapy in Han Chinese population. It has been known that a specific cohort or race of population influences the results of efficacy and toxicity of chemotherapeutic drugs (Nishiyama et al., 2015). In this study, we aimed to investigate the genetic associations between certain SNPs and ADRs or therapeutic effects in different cycles of treatment, specifically in Han Chinese patients with advanced NSCLC. This research will serve as an invaluable reference to future pemetrexed-based chemotherapy researches.

## Materials and Methods

### Patients Subjects

Two hundred three advanced NSCLC patients with Han Chinese origin were recruited from Shanghai to participate in this study. We collected the basic demographic and clinical information from the eligible participants before their enrollment. All the patients received either pemetrexed or pemetrexed-based platinum as their medical treatment against NSCLC progression. The patients were then divided into two groups. One group was composed of 100 patients who received only pemetrexed drug, whereas the rest of 103 patients received pemetrexed combinative with platinum. In both treatment groups, pemetrexed was provided at no less than 500-mg/m^2^ dosage (1 cycle of standard therapy dose) for each patient. The collected data included whole blood samples, demographics (age, gender, ethnicity), smoking history, family history of cancer, clinical stages, and Response Evaluation Criteria in Solid Tumors (RECIST) 2.0 grade.

### Candidate Genes Selection

Seven genes were selected from earlier relevant reports (**Supplementary Table 1**), and a number of genes that have been known to play important roles in crucial molecular functions such as DNA repair, folate metabolism, and cellular transportation of pemetrexed were included in our analysis. We then performed a genetic association analysis using a total of 16 SNP sites in the 7 genes (*SLC19A1*, *FPGS*, *MTHFR*, *DHFR*, *GGH*, *ATIC*, and *TS*).

### DNA Extraction and Genotyping

Germline genomic DNA was extracted from EDTA whole blood using Axygen Blood Genomic DNA Extraction Mini Kit. The primers were designed with Assay Design Suite 2.0 Software. We genotyped 16 SNP sites from the selected 7 genes (*TS*, *FPGS*, *GGH*, *SLC19A1*, *DHFR*, *MTHFR*, and *ATIC*). Genotyping were first detected by the MassArray System (Sequenom, San Diego, CA, USA). When the MassArray System failed to capture the genotypes of interest, TaqMan (Foster City, CA, USA) would be used to detect the genotypes instead. The genotype probes of TaqMan were provided by the reagent manufacturer. We strictly complied with the standard biosecurity and institutional safety procedures in the experiment process.

### Statistical Analysis

Based on the corresponding RECIST grade, the therapeutic effects of patients were classified into 4 categories: complete response, partial response, stable disease, and progressive disease. These patients’ conditions were severe, and the disease status was advanced. Therefore, there were no recorded complete response cases in our study when we used RECIST criteria to evaluate the therapeutic responses of advanced NSCLC patients. Responder was defined as patients with partial response of RECIST score, whereas nonresponder was defined as patients with stable disease or progressive disease of RECIST score. Five types of ADRs (skin injury, gastrointestinal ADRs, hematological ADRs, liver injury, and renal injury) that occurred within the 1 to 6 treatment cycles were assessed using the Common Terminology Criteria Adverse Events version 3.0 (December 23, 2009). The content of the Common Terminology Criteria Adverse Events included the definition standard of all kinds of ADRs. Quality control of SNP sites was performed based on the following standard: the success rate of genotypic testing of less than 90%, minor allele frequency of less than 1%, and removal of homogeneous genotyping in the total sample.

To perform association analysis of SNPs and ADRs, all the patients were divided into case and control groups according to their clinical records. Patients in the case groups were the ones who suffered from certain types of ADRs, and patients in the control group were ones without those specific symptoms of ADRs. For example, we considered patients with liver injury ADR as the case group and patients without symptoms of liver injury ADRs as the control group when we analyzed this specific ADR subtype. Association analysis of SNP sites in different drugs and cycles of treatment was performed accordingly. Hardy-Weinberg equilibrium parameter was set to be 0.05. The results were then analyzed using PLINK (version 1.70) software. A χ^2^ test (two-tailed *P* value threshold = 0.05) was performed to test if there existed any significant differences in SNP distributions between the responder and nonresponder groups in pemetrexed-treated patients. The demographic and clinical characteristics of patients were analyzed using SPSS (version 19.0, Chicago, IL, USA) (http://www.downza.cn/tags/SPSS/) and R (version 3.3.2) software (https://www.R-project.org/). According to our results, there were no variables (age, gender, smoking history, cancer history) that demonstrated any significant associations with occurrences of ADRs and therapeutic effects of pemetrexed-based therapy. Therefore, these variables were regarded as covariates in further association analysis.

## Results

### Patient Characteristics

**Figure 1:**
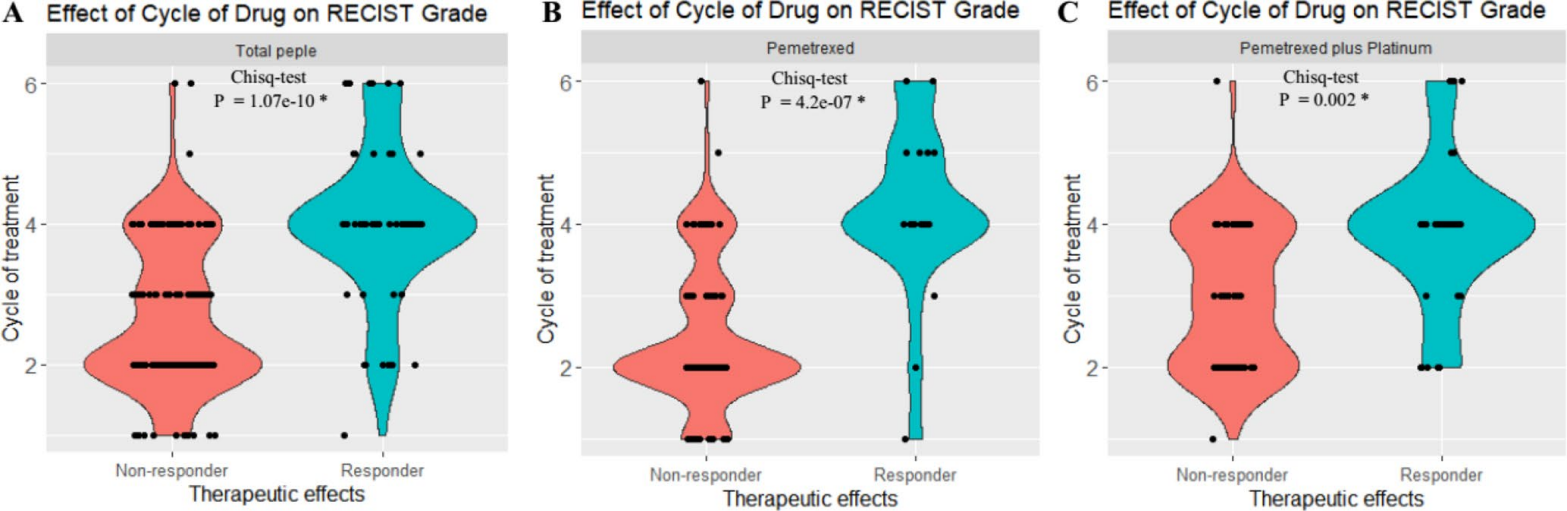
Statistical analysis revealed that there exist significant differences of the RECIST scores between the responder [partial response (*PR*)] and nonresponder groups [stable disease (SD) + progressive disease (PD)]. **(A)** Both the responder and nonresponder groups had significant differences across cycles of treatment in total patients. **(B)** Both responder and nonresponder groups had significant difference across cycles of treatment in pemetrexed. **(C)** Both responder and nonresponder groups had significant difference under different cycles of treatment in pemetrexed plus platinum. *P < 0.01. The y axis indicates the cycle of chemotreatment, and 2, 4, and 6, respectively, refer to 2, 4, and 6 cycles of chemotreatment. The x axis indicates the therapeutic effects of treatment with pemetrexed-based chemotherapy.

The demographic and clinical information of all the 203 patients is shown in **Table 1**. The patients who received single pemetrexed accounted for 49.3% of the entire sample, whereas the rest of the patients received pemetrexed-based platinum for anti-NSCLC treatment. We used R to perform the comparison and visualization of demographic and clinical data such as age, gender, family history of cancer, smoking status, ADRs, drugs, cycles of treatment, and therapeutic effects. The therapeutic effects were found to be related to the cycles of treatment in different agent groups (**Figure 1**). We found that the therapeutic effect with one to six cycles of treatment was significantly different in both groups, which was consistent with our expectation. We demonstrate that the distributions of the five types of ADRs and therapeutic effects in different cycles of treatment in **Table 1**. The results of Hardy-Weinberg equilibrium are listed in **Supplementary Table 2**.

**Table 1:** Study subject demographics and clinical status.

Characteristics		All patients		Pemetrexed		Pemetrexed plus platinum	
		Responder (n = 61)	Nonresponder (n = 142)	Responder (n = 21)	Nonresponder (n = 79)	Responder (n = 40)	Nonresponder (n = 63)
Age (y)	Median (range)	59 (23-80)	60 (29-83)	60 (46-78)	61.67 (37-83)	59 (23-80)	56.06 (29-76)
Gender	Female	27 (55.7%)	53 (37.3%)	10 (47.6%)	30 ((38.0%)	16(40.0%)	23 (36.5%)
	Male	34 ((44.3%)	89 (62.7%)	11 (52.5%)	49 (62.0%)	24 (60.0%)	40 (63.5%)
Cancer history	—	4 (6.6%)	7 (4.9%)	—	5 (6.3%)	4 (10%)	2 (3.2%)
Smoking history	Never	40 (65.6%)	90 (63.4%)	15 (71.4%)	46 (58.2%)	25 (62.5%)	44 (69.8%)
	Former	5 (8.2%)	7 (4.9%)	1 (4.8%)	5 (6.3%)	4 (10.0%)	2 (3.2%)
	Current	16 (26.2%)	45 (31.7%)	5 (23.8%)	28 (35.4%)	11 (27.5%)	17 (27.0%)
Cancer stage	IIIa	2 (3.3%)	6 (4.2%)	1 (4.8%)	5 (6.3%)	1 (2.5%)	1 (1.6%)
	IIIb	14 (23.0%)	24 (16.9%)	5 (23.8%)	12 (15.2%)	9 (22.5%)	12 (19.0%)
	IV	45 (73.8%)	112 (78.9%)	15 (71.4%)	62 (78.5%)	30(75.0%)	50 (79.4%)
Cycle of treatment	1-2	7 (11.5%)	77 (54.2%)	2 (9.5%)	52 (65.8%)	5 (12.5%)	25 (39.7%)
	1-4	48 (78.7%)	139 (97.9%)	14 (66.7%)	77 (97.5%)	34 (85.0%)	62 (98.4%)
	1-6	61 (100%)	142 (100%)	21 (100%)	79 (100%)	40 (100%)	63 (100%)
Adverse drug reaction	Total	40 (65.6%)	40 (28.2%)	11 ((52.16%)	24(30.4%)	29 (72.5%)	16 (25.4%)
	Skin injury	16 (26.2%)	3 (2.1%)	8 (38.1%)	3 (3.8%)	8 (20.0%)	—
	Gastrointestinal	22 (36.1%	4 (2.8%)	1 (4.8%)	3 (3.8%)	21 (52.5%)	1 (1.6%)
	Hematological	9 (14.8%)	20 (14.1%)	1 (4.8%)	9 (11.4%)	8 (20.0%)	11 (17.5%)
	Liver injury	7 (11.5%)	16 (11.3%)	2 (9.5%)	9 (11.4%)	5 (12.5%)	7 (11.1%)
	Renal injury	2 (3.3%)	3 (2.1%)	—	1 (1.3%)	2 (5.0%)	2 (3.2%)

All data presented in the table correspond with the time of sample acquisition. “Responder” was defined by partial response, whereas “nonresponder” was stable disease or progressive disease. Values in the parenthesis represent number or percentage, which were measured by SPSS software. 1-2 refers to 1 or 2 cycles of chemotreatment;1-4 refers to 1, 2, 3, or 4 cycles of chemotreatment; 1-6 refers to 1, 2, 3, 4, 5, or 6 cycles of chemotreatment.

### Association Analysis of ADRs and SNP Sites

We discovered several SNPs that were significantly associated with ADRs in patients who received pemetrexed or total patients as their anti-NSCLC therapy. However, no significant associations between any SNP sites and ADRs were detected in the pemetrexed plus platinum groups. The results are shown in **Table 2**.

**Table 2:** Summary of association analysis of SNP site and ADRs results.

Classification	Gene	dbSNP ID	Minor allele	Toxicity association	Cycles of treatment	OR	0.95 CI	*P*	Adjusted *P*
All patients	SLC19A1	rs1051298	C	All ADRs	1-2	2.893	1.492-5.610	0.0014	0.017
	SLC19A1	rs3788205	T	All ADRs	1-2	2.750	1.372-5.513	0.0038	0.045
	MTHFR	rs1801133	T	Hematological ADRs	1-2	3.566	1.338-9.504	0.0079	0.094
	SLC19A1	rs1051298	C	Hematological ADRs	1-2	2.581	1.019-6.537	0.0405	0.486
	SLC19A1	rs1051298	C	All ADRs	1-4	2.196	1.438-3.354	0.0002	0.003
	SLC19A1	rs3788205	T	All ADRs	1-4	1.638	1.024-2.619	0.0386	0.502
	SLC19A1	rs1051298	C	Liver injury	1-4	2.724	1.407-5.273	0.0022	0.029
	ATIC	rs12995526	T	Gastrointestinal ADRs	1-4	2.081	1.087-3.982	0.0246	0.320
	SLC19A1	rs1051298	C	All ADRs	1-6	1.927	1.287-2.885	0.0014	0.019
	SLC19A1	rs3788205	T	All ADRs	1-6	1.616	1.03-2.536	0.0360	0.503
	SLC19A1	rs1051298	C	Liver injury	1-6	2.595	1.366-4.931	0.0028	0.039
	ATIC	rs12995526	T	Gastrointestinal ADRs	1-6	2.003	1.071-3.747	0.0274	0.384
	GGH	rs11545077	A	Gastrointestinal ADRs	1-6	1.976	1.034-3.776	0.0366	0.512
Pemetrexed-treated patients	SLC19A1	rs1051298	C	All ADRs	1-2	3.143	1.370-7.208	0.0059	0.071
	SLC19A1	rs1051298	C	All ADRs	1-4	2.340	1.250-4.378	0.0072	0.094
	SLC19A1	rs1051298	C	Liver injury	1-4	3.863	1.410-10.58	0.0056	0.072
	FPGS	rs10987740	G	Hematological ADRs	1-4	2.644	0.967-7.228	0.0512	0.665
	SLC19A1	rs1051298	C	All ADRs	1-6	2.243	1.240-4.059	0.0071	0.099
	SLC19A1	rs3788205	T	All ADRs	1-6	1.923	1.029-3.593	0.0390	0.546
	SLC19A1	rs1051298	C	Liver injury	1-6	3.466	1.345-8.934	0.0071	0.099

1-2 refers to 1 or 2 cycles of chemotreatment; 1-4 refers to 1, 2, 3, or 4 cycles of chemotreatment; 1-6 refers to 1, 2, 3, 4, 5, or 6 cycles of chemotreatment. 0.95 CI: 95% confidence intervals of the OR.

In patients who received only pemetrexed, SNP rs1051298 (c.*746C > T) in *SLC19A1* was shown to be related to increased risk of all ADRs in different cycles of therapy groups (1-2 cycles: *P* = 0.0059, odds ratio (OR) = 3.143; 1-4 cycles: *P* = 0.0072, OR = 2.340; 1-6 cycles: *P* = 0.0071, OR = 2.243). In particular, SNP rs1051298 was found to be closely associated with liver injury (1-4 cycles: *P* = 0.0056, OR = 3.863; 1-6 cycles: *P* = 0.0071, OR = 3.466). This SNP rs1051298 was identified to be significant again in the analysis on all 203 advanced NSCLC patients, thus further validating our discovery. We also found that 1 or more G alleles increased the risk of severe hematological ADRs in patients who carried *FPGS* variant rs10987740 (2311G > A). However, SNP rs10987740 itself failed to pass the significance thresholds (1-4 cycles: *P* = 0.0512, OR = 2.644) in its ADR association analysis.

In total patients including those who received pemetrexed and pemetrexed plus platinum, 2 *SLC19A1* polymorphism variants (rs1051298 and rs3788205) appeared to be significantly related to ADRs. Single-nucleotide polymorphism rs1051298 was found to be associated with all the tested ADRs in different treatment cycles. In other words, we discovered that the SNP rs1051298 was significantly related to all ADRs in cycles 1 to 2 (*P* = 0.0014, OR = 2.893), cycles 1 to 4 (*P* = 0.0002, OR = 2.196), and cycles 1 to 6 (*P* = 0.0014, OR = 1.927). The SNP rs1051298 on *SLC19A1* was shown to be significantly associated with liver injury ADR in both cycles 1 to 4 (*P* = 0.0022, OR = 2.724) and cycles 1 to 6 (*P* = 0.0028, OR = 2.595). The associations between SNP rs1051298 and ADRs remained statistically significant after Bonferroni and false discovery rate multiple-testing correction. Additionally, the variant rs1051298 is associated with hematological ADRs at 1 to 2 cycles of treatment (*P* = 0.0405, OR = 2.581). The other polymorphism on *SLC19A1*, rs3788205, also appeared to be related to all ADRs (1-4 cycles: *P* = 0.0386, OR = 1.638; 1-6 cycles: *P* = 0.0360, OR = 1.616). The SNP rs12995526 (c.815-102T > C), located in the *ATIC* gene, was found to be associated with severe gastrointestinal ADRs in 1 to 4 cycles (*P* = 0.0246, OR = 2.081) and 1 to 6 cycles (*P* = 0.0274, OR = 2.003). We also discovered that GGH variant rs11545077 (c.91G > T p. Ala31Thr) was significantly associated with increased risk of severe gastrointestinal ADRs in 1 to 6 cycles (*P* = 0.0366, OR = 1.976). At last, SNP rs1801133 (665C > T) in the *MTHFR* gene was shown to be significantly associated with hematological ADRs in 1 to 2 cycles of pemetrexed-based treatment (*P* = 0.0079, OR = 3.566).

### Association Analysis of Therapeutic Effects and SNP Sites

Two *ATIC* polymorphisms, rs3821353 (c.815-294G > T) and rs12995526 (c.815-102T > C), were found to be significantly associated with the therapeutic effects in 1 to 4 cycles of treatment with only pemetrexed (**Table 3**). Although SNP rs3821353 in *ATIC* appeared to be significantly associated with the therapeutic effects to pemetrexed (*P* = 0.041), this significance did not persist when we tested the genotype difference between the responder and nonresponder group using χ^2^ test (*P* = 0.092). At least 1 T allele in another SNP rs12995526 was significantly related to higher therapeutic effects compared with G allele in 1 to 4 treatment cycles (*P* = 0.003, adjusted *P* by Bonferroni method = 0.044). This variant rs12995526 in *ATIC* remained significant using χ^2^ analysis of genotype (*P* = 0.001).

**Table 3:** Summary of association analysis between SNP site and therapeutic effects in pemetrexed-treated patients result.

Gene	dbSNP ID	Minor allele	Cycles of treatment	OR	*P*	Adjusted *P*	Phenotype	Genotype (number)			*P*
*ATIC*	rs3821353	G	1-4	2.446	0.041	0.529	Responder	AA(5)	AG(7)	GG(1)	0.092
							Nonresponder	AA(11)	AG(39)	GG(20)	
*ATIC*	rs12995526	T	1-4	3.606	0.003	0.044	Responder	TT(1)	TC(9)	CC(3)	0.001
							Nonresponder	TT(5)	TC(14)	CC(52)	
*GGH*	rs11545077	A	1-4	0.149	0.037	0.478	Responder	AA(0)	AG(1)	GG(12)	0.081
							Nonresponder	AA(2)	AG(26)	GG(43)	
*ATIC*	rs12995526	T	1-6	2.081	0.059	0.827	Responder	TT(1)	TC(11)	CC(9)	0.020
							Nonresponder	TT(6)	TC(16)	CC(57)	
*GGH*	rs11545077	A	1-6	0.092	0.0045	0.064	Responder	AA(0)	AG(1)	GG(20)	0.005
							Nonresponder	AA(2)	AG(29)	GG(48)	

1-2 refers to 1 or 2 cycles of chemotreatment; 1-4 refers to 1, 2, 3, or 4 cycles of chemotreatment; 1-6 refers to 1, 2, 3, 4, 5, or 6 cycles of chemotreatment. Phenotype: Using pemetrexed treatment for advanced NSCLC patients was response or nonresponse.

At last, SNP rs11545077 (c.91G > T p. Ala31Thr) in the *GGH* gene was found to be significantly associated with the therapeutic effects in the patient who received 1 to 6 cycles of pemetrexed treatment (*P* = 0.0045). This SNP remained to be significant in χ^2^ analysis of its genotype (*P* = 0.005).

## Discussion

Pemetrexed has been regarded as the first- or second-line conservative treatment for advanced NSCLC patients (Scagliotti et al., 2008; Rudin et al., 2013; Franchina et al., 2014). Many studies have been performed to search for biomarkers of pemetrexed response using non–Han Chinese patients. However, only a small number of studies focused specifically on the performance of Han Chinese patients with advanced NSCLC. Therefore, we conducted this study using 203 Han Chinese patients with advanced NSCLC in order to reveal the hidden biomarkers of therapeutic effects specific to this race. In addition, we also investigated the influence of other demographic, clinical, and pharmacogenomic components on therapeutic response of pemetrexed on Han Chinese NSCLC patients.

The results of this study successfully revealed that two novel SNPs (rs1051298 and rs3788205) in the *SLC19A1* gene had significant associations with the risk of ADR occurrences in advanced NSCLC patients. We discovered that SNP rs1051298 was particularly significantly related to the increased risk of severe reaction of hepatotoxicity in patients who received pemetrexed treatment. Earlier reports demonstrated that this SNP rs1051298 in the *SLC19A1* gene affected the overall survival and progress-free survival of advanced NSCLC patients who received pemetrexed plus platinum treatment (Yee et al., 2010; Corrigan et al., 2014). On the other hand, however, our study is the first where SNP rs3788205 in *SLC19A1* was identified as a genetic marker of increased risk of ADRs in advanced NSCLC patients who received pemetrexed plus platinum treatment. The significance of rs3788205 has been found to be associated only with overall survival of small cell lung cancer patients who accepted pemetrexed carboplatin therapy in a previous study (Smit et al., 2012). This absence of earlier discovery of the important role of rs3788205 in pemetrexed response may be explained by the unique genetic background of the Han Chinese patient sample used in our study. Earlier pemetrexed studies were mainly performed on non–Han Chinese patients and may not be suitable to apply on Han Chinese patients. It is possible that this newly found SNP was a genetic signature of pemetrexed response that occurred only in Han Chinese NSCLC patients.

We also identified and validated a previously discovered SNP rs1801133 (665C > T) in the *MTHFR* gene. It was shown to increase the occurrences of hematological ADRs in 1 to 2 cycles of the pemetrexed therapy group. This result was supported by a number of previous studies that have discovered a correlation between *MTHFR* variants and the efficacy or toxicity and survival time of advanced NSCLC patients who received pemetrexed plus platinum treatment (Smit et al., 2009; Li et al., 2013; Krawczyk et al., 2014; Li et al., 2014; Ding et al., 2017). It was noteworthy that SNP rs1801133 in the *MTHFR* gene has been reported to be associated with decreased risk of NSCLC in thrombocytopenia (Li et al., 2014). Because thrombocytopenia was a subtype of hematological ADRs, it suggests that SNP rs1801133 was closely related to hematological ADRs. All these literatures mentioned above were consistent with our discovery of the association between SNP rs1801133 and ADRs to pemetrexed.

At last, our results demonstrate that SNP rs12995526 (c.815-102T > C) in the *ATIC* gene and a newly discovered SNP rs11545077 (c.91G > T) in the *GGH* gene were significantly associated with both ADRs and therapeutic effects in pemetrexed-treated patients. It was suspected that ADRs and therapeutic effects to pemetrexed treatment share genetic basics, resulting in similar response when the variants appeared. Earlier studies have shown that different polymorphisms on the *GGH* and *ATIC* were both related to ADRs and survival time (Adjei et al., 2010b; Corrigan et al., 2014; Woo et al., 2015). In addition, both *GGH* and *ATIC* genes decrease the activity of pemetrexed, which lead to reduced toxicity in terminal stage (Wang et al., 2014; Fukuda et al., 2016). Together, these results suggest that the functions of the *GGH* and *ATIC* genes may be correlated, and they were both essential for therapeutic response of pemetrexed-based treatment.

This study had several limitations. First, we have not selected all potential genes that affect the pemetrexed response in our analysis, which would miss some of the important genetic biomarkers. Second, our study was performed only on Han Chinese NSCLC patients, limiting our sample pool with only 1 ethic race. Third, certain previously identified variants, such as SNP rs10987740 in the *FPGS* gene, failed to reach statistical significance in our analysis. This may be due to our small sample size, which was not large to provide enough statistical power to capture some signals. Therefore, further investigation with a larger gene pool, larger sample size, and patients with different ethical backgrounds is necessary to validate our discoveries.

In summary, we performed a systematic genetic association study of 16 SNPs in 7 selected genes (*SLC19A1*, *FPGS*, *MTHFR*, *DHFR*, *GGH*, *ATIC*, and *TS*) and therapeutic responses of pemetrexed-based treatment (ADRs and therapeutic effects) using 203 Han Chinese patients with advanced NSCLC. Our results suggest that 5 SNPs (rs1051298, rs3788205, rs1801133, rs12995526, and rs11545077) were significantly associated with ADRs to pemetrexed. In addition, 3 SNPs (rs3821353, rs12995526, and rs11545077) were found to be significantly associated with therapeutic effects of pemetrexed. Our discoveries are of great help to novel personalized medical treatment design that minimizes ADR emergence and maximizes the therapeutic effects of pemetrexed in Han Chinese patients.

## Ethics Statement

The whole process of patient recruitment and information collection was approved by Shanghai Ethical Committee of Human Genetic Resources. We strictly followed the guidelines and regulations of the committee to collect the clinical information from eligible participants. All the participants signed their informed consent by themselves in this study.

## Author Contributions

DZ analyzed the data and wrote this manuscript. LC, DZ, and XZ performed the experiments and visualization. JM, ML, and WZ aided in processing the data. XZ, LH, and GL aided in the collection of the materials. LC, MW, CZ, and JZ helped to revise this manuscript. SQ, ZW, and JZ, the corresponding authors, conceived and designed the experiments.

## Funding

This research was funded by a Shanghai "Rising Stars of Medical Talent" Youth Development Program (Clinical Pharmacist Program, 2017), the 863 Program (2012AA02A515,2012AA021802), the National Nature Science Foundation of China (81773818,81273596,30900799,81671326), National Key Research, Development Program (2016YFC0905000, 2016YFC0905002, 2016YFC1200200, 2016YFC0906400), the 4th Three-Year Action Plan for Public Health of Shanghai (project no.: 15GWZK0101), Shanghai Pujiang Program (17PJD020), and Shanghai Key Laboratory of Psychotic Disorders (13dz2260500).

## Conflict of Interest Statement

The authors declare that the research was conducted in the absence of any commercial or financial relationships that could be construed as a potential conflict of interest.
