# Supplementary material for: Discovery of Novel Biomarkers of Therapeutic Responses in Han Chinese Pemetrexed-Based Treated Advanced NSCLC Patients
Source: Front Pharmacol. 2019 Aug 23;10:944. doi: 10.3389/fphar.2019.00944 (PMC6716463; doi:10.3389/fphar.2019.00944)
Supplement: Supplementary file 1 [file Table_1.docx]

| **Gene** | **SNP ID** | **Results** | **Study participants** | ***P* value** | **Reference** |
| --- | --- | --- | --- | --- | --- |
| *GGH* | rs11545078 | T allele was a protective factor grade 3 or 4 toxicity | 136 | 0.018 | (Corrigan et al., 2014a) |
| *GGH* | rs3780126 | T allele causative of increased grade Ⅲ type lymphodpaenia | 48 | 0.030 | (Adjei et al., 2010a) |
| *DHFR* | rs1650697 | CT or TT genotypes increased incidence of grade 3 or 4 toxicity | 136 | 0.034 | (Corrigan et al., 2014a) |
| *DHFR* | rs442767 | CC genotype increased fatigue | 90 | 0.008 | (Jung et al., 2013) |
| *MTHFR* | rs1801133 | TT genotype had more longer overall survival | 208 | 0.016 | (Tiseo et al., 2012) |
| *MTHFR* | rs1801131 | AC/CC genotype related to survival time | 65 | 0.001 | (Chen et al., 2010) |
| *SLC19A1* | rs3788189 | TT genotype had less shorter survival | 38 | 0.026 | (Dy et al., 2014) |
| *SLC19A1* | rs1051298 | One or more copies of T allele had increased risk of death | 136 | 0.016 | (Corrigan et al., 2014a) |
| *SLC19A1* | rs914232 | AA genotypes were significantly associated with less shorter OS | 136 | 0.007 | (Corrigan et al., 2014a) |
| *SLC19A1* | rs2838958 | TT genotype had more longer OS | 54 | 0.030 | (Adjei et al., 2010b) |
| *FPGS* | rs138322374 | GA genotype risk of high ALT | 54 | 0.070 | (Adjei et al., 2010b) |

**Supplementary Table 1：Summary of known SNPs that connect to responses to pemetrexed treatments**
