# Supplementary material for: Discovery of Novel Biomarkers of Therapeutic Responses in Han Chinese Pemetrexed-Based Treated Advanced NSCLC Patients
Source: Front Pharmacol. 2019 Aug 23;10:944. doi: 10.3389/fphar.2019.00944 (PMC6716463; doi:10.3389/fphar.2019.00944)
Supplement: Supplementary file 2 [file Table_2.docx]

**Supplementary Table2：The relevant results of Hardy-Weinberg equilibrium**

| **SNP** | **TEST** | **Minor allele** | **Genotype counts** | **Observed heterozygosity** | **Expected heterozygosity** | | | **HWE**  **P-value** |
| --- | --- | --- | --- | --- | --- | --- | --- | --- |
| rs1801133 | ALL | T | 30/94/78 | 0.465 | | 0.472 | 0.882 | |
| rs1801133 | AFF | T | 13/37/29 | 0.468 | | 0.480 | 0.818 | |
| rs1801133 | UNAFF | T | 17/57/49 | 0.463 | | 0.466 | 1.000 | |
| rs3821353 | ALL | G | 44/111/47 | 0.550 | | 0.500 | 0.205 | |
| rs3821353 | AFF | G | 22/42/16 | 0.525 | | 0.497 | 0.822 | |
| rs3821353 | UNAFF | G | 22/69/31 | 0.566 | | 0.497 | 0.150 | |
| rs12995526 | ALL | T | 9/74/120 | 0.365 | | 0.351 | 0.690 | |
| rs12995526 | AFF | T | 4/34/42 | 0.425 | | 0.387 | 0.564 | |
| rs12995526 | UNAFF | T | 5/40/78 | 0.325 | | 0.324 | 1.000 | |
| rs371824049 | ALL | A | 0/4/192 | 0.020 | | 0.020 | 1.000 | |
| rs371824049 | AFF | A | 0/1/76 | 0.013 | | 0.013 | 1.000 | |
| rs371824049 | UNAFF | A | 0/3/116 | 0.025 | | 0.025 | 1.000 | |
| rs12681874 | ALL | T | 34/96/70 | 0.480 | | 0.484 | 0.885 | |
| rs12681874 | AFF | T | 13/36/31 | 0.450 | | 0.475 | 0.642 | |
| rs12681874 | UNAFF | T | 21/60/39 | 0.500 | | 0.489 | 0.854 | |
| rs11545077 | ALL | A | 5/71/127 | 0.350 | | 0.319 | 0.269 | |
| rs11545077 | AFF | A | 0/33/47 | 0.413 | | 0.327 | 0.033 | |
| rs11545077 | UNAFF | A | 5/38/80 | 0.309 | | 0.314 | 0.779 | |
| rs12379987 | ALL | T | 17/89/97 | 0.438 | | 0.422 | 0.739 | |
| rs12379987 | AFF | T | 6/40/34 | 0.500 | | 0.439 | 0.308 | |
| rs12379987 | UNAFF | T | 11/49/63 | 0.398 | | 0.411 | 0.826 | |
| rs10987740 | ALL | G | 41/104/57 | 0.515 | | 0.497 | 0.672 | |
| rs10987740 | AFF | G | 17/41/22 | 0.513 | | 0.498 | 1.000 | |
| rs10987740 | UNAFF | G | 24/63/35 | 0.516 | | 0.496 | 0.717 | |
| rs1544105 | ALL | G | 19/92/92 | 0.453 | | 0.435 | 0.630 | |
| rs1544105 | AFF | G | 7/39/34 | 0.488 | | 0.443 | 0.456 | |
| rs1544105 | UNAFF | G | 12/53/58 | 0.431 | | 0.430 | 1.000 | |
| rs10106 | ALL | A | 26/80/94 | 0.400 | | 0.442 | 0.200 | |
| rs10106 | AFF | A | 11/32/35 | 0.410 | | 0.453 | 0.453 | |
| rs10106 | UNAFF | A | 15/48/59 | 0.393 | | 0.435 | 0.300 | |
| rs4451422 | ALL | A | 19/90/89 | 0.455 | | 0.438 | 0.630 | |
| rs4451422 | AFF | A | 7/37/33 | 0.481 | | 0.443 | 0.608 | |
| rs4451422 | UNAFF | A | 12/53/56 | 0.438 | | 0.434 | 1.000 | |
| rs12483761 | ALL | G | 0/5/198 | 0.025 | | 0.024 | 1.000 | |
| rs12483761 | AFF | G | 0/3/77 | 0.038 | | 0.037 | 1.000 | |
| rs12483761 | UNAFF | G | 0/2/121 | 0.016 | | 0.016 | 1.000 | |
| rs1051298 | ALL | C | 44/93/66 | 0.458 | | 0.494 | 0.320 | |
| rs1051298 | AFF | C | 23/41/16 | 0.513 | | 0.496 | 0.825 | |
| rs1051298 | UNAFF | C | 21/52/50 | 0.423 | | 0.472 | 0.254 | |
| rs3788205 | ALL | T | 16/72/115 | 0.355 | | 0.381 | 0.356 | |
| rs3788205 | AFF | T | 10/30/40 | 0.375 | | 0.430 | 0.297 | |
| rs3788205 | UNAFF | T | 6/42/75 | 0.342 | | 0.343 | 1.000 | |
